# Contextual factors that influence adoption and sustainment of self-management support in cancer survivorship care: a practical application of theory with qualitative interviews

**DOI:** 10.1136/bmjqs-2024-017561

**Published:** 2024-11-13

**Authors:** Nickola Pallin, John Browne, Roisin Connolly, Josephine Hegarty, Sheena McHugh

**Affiliations:** 1 School of Public Health, University College Cork, Cork, Ireland; 2 College of Medicine & Health, University College Cork, Cork, Cork, Ireland; 3 CUH/UCC Cancer Centre, Cork University Hospital, Cork, Cork, Ireland; 4 School of Nursing and Midwifery, University College Cork College of Medicine and Health, Cork, Ireland

**Keywords:** Health services research, Implementation science, Health policy

## Abstract

**Background:**

Self-management support (SMS) is a recommended component of cancer survivorship care that improves health-related quality of life and reduces healthcare utilisation. However, widespread implementation has been difficult to achieve, with a gap in the literature on system-wide implementation efforts. This study examines contextual factors perceived to influence SMS adoption and sustainment in cancer centres in the Republic of Ireland.

**Method:**

Semistructured interviews were conducted with 47 key informants from 20 cancer organisations across community and hospital settings. Participants were asked to report the level of adoption and sustainment of SMS at their organisation. This information was used to categorise organisations as low, medium or high implementers. We conducted cross-case analysis following the principles of Framework Analysis. Using the Consolidated Framework for Implementation Research as a menu of constructs, we examined factors influencing adoption and sustainment and variation in levels of implementation.

**Results:**

National policy, external accreditation, external financing opportunities and the presence of champions in organisations are influential early in the implementation process driving adoption. Healthcare provider-led programmes and evidence of SMS improving patient outcomes and aligning with an organisation’s priorities are necessary to secure buy-in, particularly among senior leadership. An organisational culture of entrepreneurship enables adoption and sustainment, with resources and a culture supporting staff well-being enabling sustainment.

**Conclusion:**

While national policy is a driver, additional factors related to programme attributes and local contextual features such as the presence of champions, organisational readiness and culture influence implementation. The results may be used for future evaluations of SMS implementation in cancer survivorship care and to inform the development of tailored implementation strategies.

**WHAT IS ALREADY KNOWN ON THIS TOPIC**

- Internationally, widespread implementation of self-management support (SMS) programmes for cancer survivors has been difficult to achieve, despite evidence of effectiveness and consensus that such programmes are an essential part of long-term recovery.
- While previous studies have identified barriers and enablers of individual SMS programmes at a local level, few have studied national implementation efforts.
- As a consequence, there is little information on how the roll out of SMS can be optimised across organisations and at a national level and what factors differentiate between organisations with varying levels of implementation.

**WHAT THIS STUDY ADDS**

- Using the Consolidated Framework for Implementation Research as a foundation menu of constructs, this study presents a framework depicting the contextual factors perceived to be most relevant for adoption and sustainment of SMS in cancer survivorship care.
- Findings provide insight into factors that differentiate between organisations with high and low levels of implementation.

**HOW THIS STUDY MIGHT AFFECT RESEARCH, PRACTICE OR POLICY**

- Findings provide a basis to inform the tailoring of implementation strategies at national and local levels to support widespread adoption and sustainment of national cancer survivorship programmes.

## Introduction

 With improvements in diagnostics and treatments, more people are living with and beyond cancer.^1^ However, many cancer survivors experience long-term physical and psychosocial morbidity after treatment.^2^ This has led to the introduction of cancer survivorship programmes in many countries.^3^,^7^ These focus on monitoring and managing symptoms with the aim of reducing morbidity, promoting health and preventing recurrence after the acute phase of treatment.^8 9^ International organisations such as the WHO and European Commission recommend the implementation of cancer survivorship care.^10^,^14^ One element of cancer survivorship care is self-management support (SMS),^10^,^1215^ which is defined as the ‘systematic provision of education and supportive interventions by health care staff to increase patients’ skills and confidence in managing their health problems, including regular assessment of progress and problems, goal setting, and problem-solving support’.^16^ SMS programmes aim to enable patients living with a long-term condition to work collaboratively with healthcare providers to manage their own health and well-being to improve health outcomes and reduce health services utilisation.^17^,^19^ Programmes include information provision and online courses, and extend beyond didactic instructional approaches to include behaviour change interventions and coaching by healthcare professionals or trained peers to enhance patients’ self-efficacy and self-management skills.^17^,^19^ Despite evidence of effectiveness and international recommendations, widespread implementation of SMS in cancer survivorship care has been difficult to achieve.^1520^,^25^ One study evaluated the adoption and implementation of a digital SMS programme among 65 hospitals in the Netherlands and the adoption rate was 31%.^25^ In adopter hospitals, the programme was offered to 72% of patients by healthcare providers.^25^ Studies on SMS implementation have focused on single programmes in local settings,^23 25 26^ with a gap in the literature on system-wide initiatives. Few studies have assessed how local organisations react to and act on national policy guidance. There is also limited research on what is needed to implement national policy recommendations in the context of existing local initiatives which may be disrupted. Understanding this is important as there are unique factors surrounding policy implementation that differ from local implementation initiatives.^27^

In Ireland, the 2017–2026 National Cancer Strategy recommended the implementation of survivorship programmes including SMS.^28^ Programmes have been adopted across different regions and care settings,^29^ and through a multisite qualitative study, we examined the contextual factors influencing adoption and sustainment of survivorship programmes that incorporate SMS in an Irish healthcare setting.^30^ Studies of implementation context often generate isolated lists of barriers and enablers without explaining how or why these factors are important and at what stage of implementation.^31^ Our study responds to the call for ‘theoretically informative’ improvement research^32^ by presenting a framework of contextual factors perceived to influence service level implementation of SMS programmes and generating potentially testable explanations for variation in implementation.

## Methods

A qualitative study design was used, involving semistructured interviews with key informants across 20 cancer services including hospitals and community cancer support centres in the Republic of Ireland. Reporting follows the Standards for Reporting Qualitative Research guidelines^33^ (online supplemental file 1).

## Study setting

In Ireland, there are two types of public hospitals. One is funded by the state and managed by the Health Service Executive (HSE), and the other type is state funded managed by private bodies or charities known in Ireland as ‘voluntary hospitals’. Despite the different management and ownership models, all public hospitals are financed primarily by Government taxation. There are also private hospitals that receive no state funding.

## Selecting centres

Ireland’s cancer services are structured around a hub and spoke model featuring eight National Cancer Control Programme designated adult cancer centres located in public hospitals as primary hubs.^28 34^ We purposively selected these eight centres because they have sufficient ‘case volumes, expertise and concentration of specialist skills, working in multidisciplinary teams to ensure the best outcome for patients’ and to ensure geographical representation.^35^ Four centres are located in HSE-managed hospitals and four are located in ‘voluntary hospitals’. We also included community cancer support centres operated by the charity sector which provide psychosocial care and SMS for cancer survivors and their family.^34^ Community cancer support centres are non-profit organisations that provide non-acute care for individuals living with and beyond cancer, their families and caregivers. There are an estimated 35 centres, operating largely on independent funding from donations and grants.^34 36^ They offer services free of charge, including information and education about cancer, psychological support through counselling, and survivorship and SMS programmes. They also offer complementary therapies such as yoga and relaxation classes, facilitate support groups for shared experiences and provide financial advice.^34 36^ These centres were identified through our advisory group who provided guidance on where SMS programmes are delivered. We also identified community cancer support centres via listings online which provide information about their services.

### Sampling and recruitment

Potential participants were invited via email using both purposive criterion and snowball sampling^37^ to select ‘information-rich cases’.^38^ We interviewed individuals who delivered programmes or care that focused on supporting cancer survivors in developing at least one self-management task (ie, medical management, role management or emotional management)^19^ or had a role in referring patients to SMS services. Across organisations, we first contacted management seeking permission to conduct the study and they identified individuals directly involved in delivering SMS. These participants were invited to participate and then asked to suggest other individuals who could contribute to the study. Interested participants contacted NP by email or phone. Participation was voluntary, and all interviewees provided informed consent. Sample size was informed by the principles of information power whereby the more information the sample holds, the lower number of participants required.^39^ Information power focuses on the depth and relevance of the data, rather than focusing on a set number of participants or the absence of new themes to guide sample sizes.^39^ The research focus was narrow, the interviews and quality of dialogue between the interviewer and participant were in depth and averaged 50 min, the topic guide was informed by pre-existing literature and a theoretical framework. Purposive sampling was also used to ensure participants had in-depth knowledge and experience relevant to the research question.^39^

### Expert advisory group

Our expert advisory group reviewed the topic guide, which was refined accordingly. For example, one member of our patient and public advisory group who has experience in healthcare quality improvement in the Irish setting suggested exploring the influence of hospital ownership on implementation. The advisory group also helped frame the research question, provided feedback on data collection procedures and supported recruitment. The group included five individuals with lived experience of cancer and members from the National Cancer Control Programme cancer survivorship team. Our research team included researchers and clinicians with expertise in cancer care and survivorship delivery, quality improvement, implementation science and health services research.

### Data collection procedures

From June 2022 to March 2023, interviews were conducted in person, over telephone or online by NP, a radiation therapist with experience of qualitative data collection. Interviews were audio-recorded and transcribed by NP or a professional transcription service and anonymised by NP. Topic guide questions were structured around the updated Consolidated Framework for Implementation Research (CFIR) which sets contextual influences on implementation (n=48 constructs).^40^ The topic guide (online supplemental file 2) focused on CFIR constructs that had been identified as potentially relevant to SMS implementation in previous research.^2325 41^,^43^ The topic guide also elicited descriptions of programme adoption and sustainment.^44^

### Analysis

**Figure 1:**
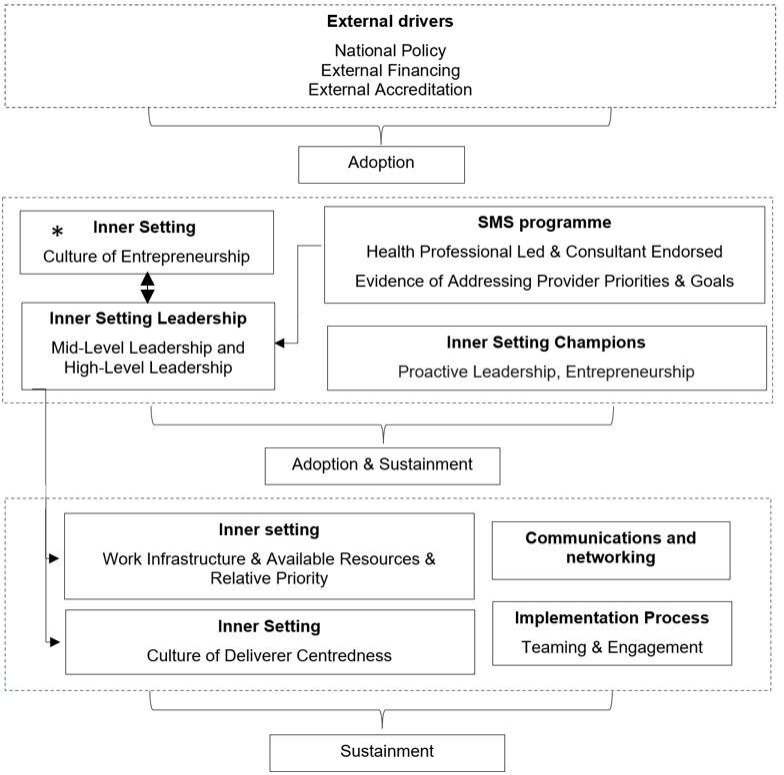
Framework depicting contextual factors influencing implementation. *Strengthen the effects of enabling factors (eg, champions)

We used a combined inductive-deductive coding approach following the Framework Analysis principles.^45^ First, we developed qualitative definitions for non-adoption, adoption, sustainment and non-sustainment based on published definitions (table 1).^44^ Based on participant reporting of adoption and sustainment, we classified organisations as low, medium or high implementing sites. Organisations with sustained programmes were classified as high implementing, those with adopted programmes as medium, and those with no programmes as low implementing. This classification of organisations allowed us to compare ‘cases’ to understand the most relevant factors contributing to these differences. To identify contextual factors, we then coded transcripts inductively and mapped these codes to the CFIR framework.^40^ Initially five transcripts were double‐coded (independently by NP and SMH). Coders discussed any discrepancies and agreed on the most appropriate CFIR construct to develop an analytical framework to code the remaining transcripts using NVivo. Codes relating to a given contextual factor (eg, champions) were grouped together and summarised in a matrix with contextual factors as rows and organisations (cases) as columns (see online supplemental file 3). In some organisations (n=9), the sample had one participant. While others (n=11) had multiple interviews conducted within the same organisation. For organisations with multiple participants, their perspectives were integrated. Findings were compared iteratively across cases to understand how and under what circumstances factors influence adoption and sustainment.^45^ See onlinesupplemental files 3  4 for more detail and supporting quotes. Relationships between contextual factors were also explored across and between cases.^46^ This enabled us to present a theory-informed framework of contextual factors most relevant for adoption or sustainment or both and the relationship between contextual factors (figure 1).^47^ To ensure trustworthiness, interpretations were periodically discussed among the research team and presented to our expert advisory group.

**Table 1:** Qualitative definitions of implementation outcomes and participant quotes for perceived adoption and sustainment

Implementation outcome category	Definition of implementation outcome	Published definitions from the literature	Example of participant quotes used to determine perceived extent of adoption and sustainment
**Non-adoption**	Non-adoption of SMS programme. These organisations had no SMS programme adopted. In these organisations health providers often refer or direct cancer survivors to SMS outside of organisation.	*Adoption is defined as ‘the intention, initial decision, or action to try or employ an innovation or evidence-based practice’*.^44^	*Would highlight the resources that are available to them in the community. Would recommend they all touch base with their local cancer support centre, I highlight the Cancer Thrive and Survive programme. I advise them of the nearest two cancer centres*.
**Adoption**	SMS programme adopted with initial decision, or action to try implement a programme. Or a SMS programme is supported through temporary funding or external financing for adoption but not permanently funded.		*A grant call and we applied for that and that was the catalyst really. So, without that, without us getting that grant this wouldn’t be in (organisation*).
**Non-sustainment**	SMS programme not maintained within a service setting’s ongoing, stable operations, including interruptions or gaps in delivery. This includes programme cessation following end of temporary external financing.	*Sustainability is defined as ‘the extent to which a newly implemented treatment is maintained or institutionalised within a service setting’s ongoing, stable operations’*.^44^	*We’re in the process, we haven’t done one this year, we delivered two programmes before 2020*.
**Sustainment**	SMS programme maintained or institutionalised within a service setting’s ongoing, routine delivery of care. This includes necessary resource allocation for sustained delivery.		*It’s clinical service and continuously being delivered as part of standard of care*.

Qualitative definitions for non-adoption, adoption, sustainment and non-sustainment based on published definitions of implementation outcomes.^44^

SMS, self-management support.

## Results

We interviewed 47 key informants (average key informants per site 2.4 (range 1–7)) across 20 organisations. These included 8 hospitals and 12 community cancer support centres. Participants represented local level management, trained peer leaders and healthcare providers. Healthcare providers included nurses, psychiatrists, psychologists, occupational therapists, physiotherapists, dietitians, social workers, surgeons and medical oncologists (table 2). Perceived implementation level varied across organisations, with eight high implementing sites, nine medium and three low implementing sites (table 2). Interviews lasted approximately 50 min (range 30–120 min).

Figure 1 depicts the most relevant factors influencing perceived adoption and sustainment, and the relationship between factors. Factors are grouped into three categories: drivers of adoption, factors influencing both adoption and sustainment and factors influencing only sustainment. For example, policy and external financing were influential early in the implementation process driving adoption. Whereas, champions, leadership support, a culture of entrepreneurship within organisations and characteristics of the programme influenced both adoption and sustainment. The arrows illustrate the direction and nature of the relationships between factors, showing how one factor can influence others in the implementation process. For example, some have contingent relationships with adoption and sustainment. For example, the allocation of resources for implementation was perceived to be contingent on leadership buy-in and support. There were also reciprocal relationships between contextual factors, for example organisational culture and leadership interacted with each other to drive adoption and sustainment. An entrepreneurial culture influenced leadership support by fostering positive attitudes among leadership towards implementing new innovations. While leadership actions shaped and reinforced the culture, creating an environment that either supported or impeded the innovation.

**Table 2:** Number and role of participants within high, medium and low SMS implementing organisations

Organisations (n=20)																			
**High**								**Medium**									**Low**		
**1**	**2**	**3**	**4**	**5**	**6**	**7**	**8**	**9**	**10**	**11**	**12**	**13**	**14**	**15**	**16**	**17**	**18**	**19**	**20**
**Participants**
Managers (n=5)	1	1	1	1	1
Nurses (n=13)	1	1	2	1	1	1	2	1	1	2
Psychiatrists (n=2)	1	1
Psychologists (n=4)	2	1	1
Counsellors (n=2)	1	1
Occupational therapists (n=1)	1
Physiotherapists (n=5)	1	1	1	1	1
Dietitians (n=2)	2
Social workers (n=2)	1	1
Surgeons (n=1)	1
Medical oncologists (n=1)		1
Trained peer leaders (n=9)	4	1	1	1	1	1
**Total (n=47**)	**4**	**2**	**1**	**3**	**1**	**5**	**7**	**2**	**1**	**3**	**1**	**1**	**1**	**1**	**1**	**1**	**4**	**3**	**2**	**3**

Table displays implementation levels of the 20 organisations. Numbers 1 to 20 refer to each of the 20 organisations. High, medium and low refer to the level of implementation for each of the 20 organisations. Organisations with sustained programmes were high implementing (n=8), those with adopted programmes were medium (n=9) and those with no programmes adopted or sustained were low implementing organisations (n=3).

SMS, self-management support.

### Policy is a driver of adoption, but infrastructure and resources in the inner setting are necessary for sustainment

While national policy recommendations created an impetus for implementation, local conditions had to be ripe to act on those recommendations. The Irish National Cancer Strategy recommending the implementation of cancer survivorship care played a key role in legitimising and encouraging the adoption of SMS programmes across organisations. It mobilised external financial support from advocacy groups, such as research charities, and support from senior policy leadership. For example, the National Cancer Control Programme facilitated wide scale adoption of one programme called ‘Cancer Thriving and Surviving’ through funding and staff and peer-leader training. Some participants who adopted this programme described how they wanted to be part of the national adoption of this programme.

We wanted to be part of the national run of that programme as everybody else does in the country. (Participant 9, high implementing organisation)

A barrier for many healthcare providers was carrying out administrative tasks to deliver a programme alongside existing responsibilities. In low and some medium implementing organisations, programme delivery was an additional task without alignment to existing roles and duties. Adoption therefore required absorption into the current work infrastructure, without additional resources of funding, protected time and administration support.

Certainly for me, if there was more organisational support from administration, I would probably be more inclined to do it. (Participant 12, High Implementing Organisation)

Individuals in these settings acted as champions, often delivering programmes beyond their regular working hours, leading to concerns about sustainability. Some organisations developed partnerships with other organisations to acquire resources necessary for sustainment. Higher implementing organisations also had an enabling work infrastructure characterised by having leadership who provided resources to integrate administrative tasks and SMS into core operations rather than treating them as supplementary. Participants’ direct line manager also acquired additional necessary resources for sustainment including staffing, funding, protected time and physical space.

We’re so under-resourced across the board, it was a big thing for her to say, ‘try it, here’s some funding, I’ll take you out of your clinical post.’ So, someone had to fill my gap when I left, and I got this programme up and running … and her leadership and her support were key. (Participant 02, high implementing organisation)

### Accreditation, performance measurement and governance to enable adoption and continued engagement with implementation

Some participants working in hospital settings described how meeting external standards such as Organisation of European Cancer Institutes (OECI) accreditation helped formalise networks and communication between teams to drive innovation and improvements in care delivery, including survivorship care. Participants also described how performance measurement pressure, including key performance indicators and audit and feedback could motivate healthcare providers to engage with implementation and promote standardisation across organisations.

Whether that needs to be more prescriptive in our care, it needs to be measured by KPIs or through audit. I think something like that would motivate or enable better engagement. You need to see your reward for doing it. (Participant 01, low implementing organisation)

However, there was a concern that setting key performance indicators could lead to the adoption of programmes and actions to meet short-term achievable metrics without putting measures in place to identify areas for improvement and address implementation challenges. Governance frameworks and monitoring systems were therefore deemed important to standardise implementation of policy recommendations and guide monitoring and evaluation of programmes.

If you’ve got good quality management and good coordination and there’s a feedback loop, that’s very sustaining. (Participant 20, high implementing organisation)

### Providing evidence of SMS improving patient outcomes and addressing leadership priorities secures organisational buy-in

Prioritising patient needs and reporting on patient-reported outcomes by presenting data and evidence of how SMS improves cancer survivor outcomes were important for securing buy-in during the initial adoption phase. Establishing trust across stakeholder groups and gaining management and healthcare provider buy-in also relied on evidence demonstrating clear benefits in terms of effectiveness and safety.

It was very much about allowing the organisation to provide programmes that have research done into them, that have a proven track record. (Participant 36, high implementing organisation)

In addition, some interviewees unfamiliar with SMS expressed uncertainty about patient eligibility criteria. There were also concerns that patient involvement in psychosocial programmes could reinforce illness roles. Participants described the significance of patient experience feedback or testimonials to address these initial concerns, but also to motivate implementation leads to continue delivering SMS.

But once they had a couple of patients go through it, and they got feedback from their patients, they're very happy to refer. Again, because it is safe, because it is evidence based. (Participant 20, high implementing organisation)

In contrast with high implementing organisations, participants in low and medium implementing organisations described SMS and survivorship care as a low priority relative to other services, and therefore difficult to gain buy-in at management level for resource allocation. Management priorities primarily centred on reducing admissions and achieving cost savings. However, within the context of competing priorities and resource limitations, the presentation of evidence illustrating how SMS addresses the organisation’s priorities were essential for obtaining high-level management buy-in to secure resources for sustainment.

Data is everything – if you want something to progress, you need the numbers, because it decreases length of stay, there’s more cost savings for the hospital. (Participant 42, medium implementing organisation)

SMS programme credibility and trustworthiness also influenced adoption decisions. For example, health professional-led programmes were essential for securing buy-in across professions. Participants in hospitals also emphasised the importance of endorsement from oncology consultants who are senior medical oncologists in generating high-level management support for SMS.

You really don’t know where to go with hospital management. We have yet without the support of the consultants, and really the badgering from the consultants that they really need this to make their service work. (Participant 01, low implementing organisation)

However, participants expressed how differing priorities among healthcare providers can make it challenging to select appropriate evaluation metrics and present data in a way that secures buy-in.

Continued advertising of programmes including their benefits was also identified as important, as existing programmes may be overshadowed by new priorities.

### Champions with proactive leadership and entrepreneurial skills enable adoption and sustainment

Across medium and high implementing sites, there were champions actively driving adoption and sustainment, whereas such champions were not present in the low-implementation sites. Champions were individuals who actively promoted and drove the implementation of SMS across their organisation. Champions varied between those in leadership roles, including senior managers and those in healthcare provider roles such as nurses and psychologists. Across organisations, champions’ dedication fostered a bottom-up drive and commitment to implementation. In the absence of national funding opportunities, these champions demonstrated proactive leadership, and entrepreneurial skills to avail of external funding to trial a SMS programme. They also displayed enthusiasm and persuasive communication skills to develop connections and secure buy-in across various levels of influence. One champion who worked in a medium implementing organisation persevered and drove the adoption of SMS despite challenges.

I’m the driver in the service, I’m the one who keeps pushing … I might be knocked down today and tomorrow, but I’ll keep going, there’s always solutions. (Participant 41, medium implementing organisation)

In hospitals that lacked a supportive infrastructure, champions in these settings took on additional administrative tasks required to deliver SMS alongside their existing responsibilities, despite the lack of clear directives or defined roles. For example, one champion drove SMS implementation in her setting by taking the initiative to seek leadership support to adopt a programme alongside her existing responsibilities, despite uncertainty regarding responsibility for implementation among healthcare providers.

Nobody has come to me and said you need to do this, and this is what we have chosen, and this needs to be part what you’re delivering if you're delivering a survivorship service. (Participant 21, high implementing organisation)

Champions were also important for information sharing and increasing awareness of SMS among staff in their organisation. Participants described how one-off education sessions can appear formal, and ongoing working together and frequent communications from champions are more likely to build awareness of SMS and secure buy-in among healthcare staff.

You can deliver education, but I think that tends to be perceived as being formal and can be difficult to get the tone right. Whereas I think ongoing working together is what helps make things work better. (Participant 28, high implementing organisation)

### Organisational culture of entrepreneurship and addressing employee well-being affects the capacity of champions and staff to adopt and sustain programmes

A culture of entrepreneurship and innovation within organisations enabled champions to adopt and sustain SMS. This entrepreneurial culture was characterised by a set of shared attitudes, goals and practices that support entrepreneurialism. These organisations encouraged staff to take initiatives in adopting new programmes. For example, high implementing hospitals had a culture where there was freedom to initiate change and an implicit expectation to innovate and shape care delivery methods, with leadership support and less administrative pushback. Participants also described having more autonomy to shape service delivery and being able implement things quicker. In contrast, individuals in low and some medium hospitals described an unenterprising culture.

They don’t want to know what we’re doing as long as we’re showing up for work, as long as we’re practising safely, as long as there isn’t a complaint about us … it would be great if we had these performance meetings to say look, this is where I want to see, this is what I want to do. There’s none of that, it’s a pity that culture isn’t there. (Participant 41, medium implementing organisation)

In hospitals, this culture appeared to be shaped by governance and ownership, as some participants described differences between non-voluntary-led hospitals (ie, Health Service Executive (HSE) led) and ‘voluntary hospitals’.

I've worked in HSE run hospitals which are different but the (hospital) has its own board of management and it tends to prioritise innovation over a lot of other things. So, we have a certain amount of freedom in terms of how we design things. (Participant 38, medium implementing organisation)

Participants working in non-voluntary-led hospitals described processes for approval and resource allocation as slow and arduous due to administrative structures and ‘layers of management’ leading to a loss of motivation among healthcare providers to drive implementation. Strategies to support employee well-being were also important to sustain the motivation and capacity of staff to deliver programmes. Recognising the potential emotional toll associated with delivering psychosocial support and SMS, there was an emphasis on the importance of peer supervision and debriefing sessions.

Supervision has always been part of our practice, but I don’t know if it is for a lot of the other professions in this area … there’s a lot of an emotional load that comes with working in oncology, and I think if people are to be sustained in the work, and if people are also to keep their warmth and care towards patients, they have to be supported themselves as well. (Participant 15, medium implementing organisation)

Line management in high implementing organisations also fostered an environment with high staff morale by providing non-monetary incentives such as praise and recognition and bringing team members together to collaborate on tasks to implement the programme.

## Discussion

By analysing the implementation of various SMS programmes across different settings with different levels of implementation, we contribute to the understanding of the contextual factors influencing adoption and sustainment of SMS in cancer survivorship care. National policy is a driver of SMS adoption; however, it is insufficient on its own. Our findings suggest that organisations need the right internal conditions to respond, including the presence of enterprising champions, leadership buy-in, entrepreneurial culture, teamwork, networks and communications, work infrastructures and resources. In addition, enabling SMS programme attributes include healthcare provider led programmes, consultant endorsement, evidence of effectiveness in improving cancer survivor outcomes and alignment with organisational priorities. Our findings also highlight the importance of addressing multiple factors rather than isolated barriers when trying to improve SMS implementation.

We also describe potential strategies that could improve SMS implementation (table 3).^48 49^ Introducing new SMS programmes requires a shift in existing workflows, and healthcare providers spoke about conflicting priorities such as patient care and administrative responsibilities, along with limited resources as barriers. Meeting external standards was described as enabling leadership-buy-in and subsequent resource allocation and formalisation of structures and workflows. Therefore, one possible strategy to drive adoption is building SMS into the external accreditation of cancer services. Another possible strategy is the measurement of performance indicators such as number of patients who participate in SMS or number of programmes delivered within specified timeframes, with audit and feedback.^50^

**Table 3:** Strategies to address contextual factors influencing implementation of SMS

Strategy		Description
1	Mandate change	Develop policies and guidelines that prioritise SMS and outline the responsibilities of healthcare providers for implementation.
2	Change accreditation	Integrate SMS into external accreditation standards so that those who want to meet external accreditation standards are required to implement SMS.
3	Audit and feedback	Implement performance measurement using key performance indicators or audit and feedback to monitor, evaluate and modify behaviour.
4	Identify and prepare champions	Identify and support champions within the organisation who can advocate for and lead SMS implementation.
5	Recruit, designate and train for leadership	Engage health services’ leadership buy-in for resource allocation and to foster a culture of entrepreneurship and continuous quality improvement to drive innovation and enhance SMS implementation.
6	Develop and organise quality monitoring systems	Develop processes for ongoing monitoring and evaluation to identify areas for the purpose of quality assurance and improvement in SMS implementation.
7	Develop resource sharing agreements	Develop partnerships with organisations that have resources needed to implement the innovation, such as administrative support or physical space.
8	Alter incentive structures	Provide incentives to motivate staff to implement SMS.
9	Obtain and use patients/consumers and family feedback	Develop strategies to increase patient feedback on their experience of SMS.
10	Communicate with stakeholders the continued impact of the evidence-based intervention	Advertise and communicate data to external stakeholders, end-users and consumers to demonstrate the ongoing benefits, cost-effectiveness or return of investment of the innovation with continued implementation to sustain buy-in from healthcare providers and patients.

Strategies 1–9 are derived from the Expert Recommendations for Implementing Change (ERIC) compilation,^49^ while strategy 10 is derived from Nathan *et al.*^48^

SMS, self-management support.

Collecting patient-reported data and demonstrating that SMS improves cancer survivor outcomes helped to secure buy-in among leadership and healthcare providers for adoption and sustainment.^26 51^ We also found that continued advertising and promotion of SMS was important to maintain buy-in at the healthcare provider level and to stop existing programmes from being overshadowed by newer priorities. Our findings also highlight the need for champions to drive adoption and sustainment of SMS. Identifying and developing champions may be difficult in contexts that lack engaged leadership or a culture of entrepreneurship and innovation. In their study, Howell *et al*^23^ highlighted that leadership support and a culture of quality improvement enabled organisations to be more receptive to implementing SMS in Canada. A realist review found that managers who endorse and communicate their expectation for SMS motivates staff to prioritise SMS delivery.^50^ While there is limited evidence on the effectiveness of strategies for sustaining evidence-based interventions, a review identified leadership support as a commonly used strategy for public health interventions.^52^ Therefore, future efforts should consider strategies that enhance leadership buy-in for SMS implementation. This could be achieved by presenting evidence of how SMS implementation aligns with an organisation’s priorities such as reducing admissions and achieving cost savings.^52^ Strategies are also needed to cultivate an organisational culture that focuses on innovation and continuous improvement in cancer survivorship care.^53^ There is limited evidence on how to do this,^54^ but engaged leadership^54^ and a commitment to accreditation can have positive impacts on culture, performance and leadership support.^55^ Engaging clinicians from the outset may also foster a sense of ownership.^51 56^ When implementing the UK National Cancer Survivorship Initiative, national level commitment provided impetus and financial backing, but engagement of clinicians at local level was key to bringing about a cultural change. For example, ministerial approval was obtained to secure the support of senior stakeholders and clinicians were brought together to address and resolve implementation challenges.^56^

Recognising and rewarding staff efforts may also incentivise motivation and engagement.^57^ This should be supplemented with administrative support so that clinical staff have the time to focus on SMS programme implementation. Prioritising administrative efficiency may also boost staff morale and productivity as our findings highlight how a culture addressing employee well-being enables sustainment. This aligns with the ‘Quadruple Aim’ which recognises the importance of addressing the well-being of staff.^58^ In addition, it is possible that peer supervision and debriefing sessions will sustain motivation and capacity among individuals delivering SMS and reduce the emotional toll of delivering these programmes.

Although our participants did not discuss the impact of digital solutions, there is evidence that technology may enhance SMS programme delivery and patient engagement.^59^ For example, online or app-based delivery of SMS can help a programme to scale up and accommodate a growing number of users without requiring substantial resources. Digital solutions also reduce geographical and time constraints.^59^,^61^ The findings from this study can be applied to other similar contexts but strategy adaptations may be necessary to address specific local conditions, such as different organisational cultures or resource availability. For example, external accreditation and performance measurement may be influential drivers in Ireland but may not work as well elsewhere depending on local practices and regulatory frameworks. Further comparative studies are required to deepen our understanding of which contextual factors and implementation strategies have broad applicability.

### Strengths and limitations

The integration of multiple perspectives from key informants across different organisations enhances the generalisability of our findings.^30^ Our focus on identifying only the most relevant factors for implementation^30 62^ is another strength, as is our provision of testable hypotheses about implementation strategies. A limitation of our study is the lack of routinely available quantitative data on implementation outcomes. This meant we were not able to identify combinations of factors that are minimally necessary or sufficient for achieving particular targets.^63^ Implementation outcomes were reported qualitatively which lacks precision.^44^ In addition, while Ireland’s National Cancer Strategy has been supported by government funding, a limitation is the lack of specific information on national level funding and financial support for SMS, as neither public reports or interviews provided detail on the funding amounts. This leaves uncertainty about the weight of funding influence on programme adoption. In addition, while we included a large number of organisations, we had relatively few interviewees per site. However, the study was guided by the principles of information power,^39^ and key individuals who were directly involved in the implementation of SMS were purposively selected within each organisation. Therefore, even with fewer informants these participants provided in depth insights. Finally, this study did not explore the perspectives of patients and their families. Including their views could have offered greater insight into how SMS programmes align with their needs and preferences, as well as patient-level drivers of adoption and sustainment. For example, their perspective could have given more insight into ways to monitor and evaluate the effectiveness of SMS.

## Conclusion

National policy, programme attributes and local contextual features are key drivers of SMS implementation. When tailoring strategies to local contexts, multiple factors have to be addressed to improve implementation outcomes.

## Supplementary material

10.1136/bmjqs-2024-017561 online supplemental file 1

10.1136/bmjqs-2024-017561 online supplemental file 2

10.1136/bmjqs-2024-017561 online supplemental file 3

10.1136/bmjqs-2024-017561 online supplemental file 4

## Data Availability

Data are available upon reasonable request.
