# Supplementary material for: Contextual factors that influence adoption and sustainment of self-management support in cancer survivorship care: a practical application of theory with qualitative interviews
Source: BMJ Qual Saf. 2024 Nov 13;34(12):e017561. doi: 10.1136/bmjqs-2024-017561 (PMC12703244; doi:10.1136/bmjqs-2024-017561)
Supplement: online supplemental file 2 [file bmjqs-34-12-s002.pdf]

Supplementary file 2: Topic guide

Background
As you know self-management support is provision of education and supportive interventions increase patients' skills and confidence in managing their health problems.

Rationale

- We want to identify the factors influencing the approach taken to support patients to self-manage, such as intervention/programme selection and implementation approaches.
- We want to understand any barriers or facilitators to the implementation of self-management support.
- We hope that our results will inform the development of an intervention to improve the implementation, sustainability and uptake of self-management support services for cancer patients in Ireland.

Just some **general housekeeping** before we start.

- Confirm consent verbally and confirm participant has read the information leaflet.
- The interview should last approximately 40 minutes.
- If it is ok with you I will audio record the interview.
- Anything we discuss will be confidential and your identity will remain anonymous on any reports or publications. We may use direct quotes from this interview but again I stress that your name will **not** appear anywhere and neither will that of your organisation.
- Finally, you can stop the interview at any point, if you wish.
- Do you have any questions for me before we get started?

*Some questions are only applicable for those involved in implementing a programme<br><br>**Question**	**CFIR Domain**
Can you tell me a little about your role(s)?	Individuals
How is SMS delivered in your setting?<br>• Who delivers the SMS/SMS programme?<br>• How often is it run?<br>• How long has it been running?	Individuals<br>Innovation<br>Implementation Process

• How do you coordinate with other team members?<br>• What kind of space is used for the programme?	
How did you first hear about this SMS programme? How did you get involved?<br>• Were there any events that triggered plans for the delivery of SMS?<br>• Who is the driving force behind implementing this SMS programme in your setting?<br><br>*Probes: Policy, individual health professionals, University research group, Health professional group, patient group, governmental agencies, funders.*	Outer Setting<br><br>Inner Setting
What kind of resources have you received to deliver SMS?<br><br>*Probes: Training, Financial support*	Inner Setting<br><br>Implementation Process
How does SMS fit in with your role, other priorities and daily work?	Inner setting<br>Individuals
How is SMS/SMS programme communicated/advertised in your setting?	Inner Setting
Is the programme continuously being used and embedded within practice delivery?<br>• Is it part of standard of care for all patients?<br>• How do those involved make sure the programme is delivered?	Inner Setting<br>Implementation Process
Does the infrastructure (e.g. staffing) of your setting affect the implementation of SMS?<br>• Are there any infrastructure changes needed to accommodate the delivery of SMS?<br>• What needs to be considered to ensure the programme remains in place?	Intervention Characteristics<br>Inner Setting
What kind of information or evidence are you aware of that shows whether this SMS programme works/improves patients' outcomes?<br><br>*Probes: Practice guidelines/ Published literature/ Co-workers/Participant evaluation of the SMS* programme	Innovation
Now that SMS is adopted how much time and effort is required to run SMS programme?<br>• Do you think it's sustainable?<br>• Are there any changes needed to continuously deliver it?<br>• Do you have sufficient resources to continue to deliver the programme?<br><br>What would be needed to keep SMS going on a long-term basis?	Outer Setting<br>Inner Setting<br>Implementation Process
Are there other high priority cancer survivorship initiatives or activities are already happening in your setting?	Innovation

• How does this SMS programme fit with your other programmes?<br>• Does SMS take a back seat to other high-priority initiatives going on?<br>• Do you think there is a shared understanding among the professional groups and leadership about the purpose of SMS and its value?	Inner Setting
Do you think the people you work with influence implementation?<br>• How would you describe the culture of your organisation in relation to implementing SMS?<br>• Do you think this/culture influences the implementation of the intervention?<br><br>*Probe: Hospital ownership & governance*	Inner Setting<br>Individuals
**Recap**<br>Is there anything that I haven't touched on that you think is important?<br>Can you suggest other people who might be useful for us to contact?<br>**Summarise and thank participant**	
