# Supplementary material for: Contextual factors that influence adoption and sustainment of self-management support in cancer survivorship care: a practical application of theory with qualitative interviews
Source: BMJ Qual Saf. 2024 Nov 13;34(12):e017561. doi: 10.1136/bmjqs-2024-017561 (PMC12703244; doi:10.1136/bmjqs-2024-017561)
Supplement: online supplemental file 3 [file bmjqs-34-12-s003.pdf]

Supplementary file 3: Example of mapping data into matrix

	**Level of Implementation**						
	**High**			**Medium**			**Low**
	**Organisations**						
**Codes**	**Organisation 7**	**Organisation 4**	**Organisation 6**	**Organisation 11**	**Organisation 14**	**Organisation 17**	**Organisation 18**
Partnerships & connections	*We're able to do it with our local community and they can help us with admin. So that works really well. Couldn't do it without that because they set up the calls, they do the admin.* (Participant 20)	*Yes, it's through our own connections. I'd be a big believer in connecting with other organisations within our community settings, I think we're all trying to do our best and there's lots of really good spaces.* (Participant 36)	*There's no meeting space to deliver the group, so we have close links with (University). So, the last time it was run, we ran it in a room over in the (University) building).* (Participant 03,	*People are still very parochial. So, I think we have to take that global approach.* (Participant 13)	*We work very closely together; we have a good working relationship with them. So that's where it came from.* (Participant 34)		*Because we're divided by geography, so we work autonomously. It's great to see things like the NCCP who are so active and really trying to amalgamate services. I think that's definitely something I see as a bit lacking.* (Participant 06)
Communications	*You can deliver education, but I think that tends to be perceived as being formal and can be difficult to get the tone right. Whereas I*		*We talk a lot at the chemo education talks... and then I do a lot of work outside, meeting with teams, encouraging referrals, education, training staff member. We try and attend in-*			*A barrier is sharing of information, of what's going on. I think everyone has their own information, but sometimes maybe we don't share it amongst the professionals. I think the sharing*	

	think ongoing working together is what helps make things work better. (Participant 28)		*services, meetings, we did a grand rounds presentation. We have internal communications through newsletters and our internal hospital magazine, so trying to include the information in that as well, and then we have shared notes online.* (Participant 02)			*of information and knowledge between staff working in the same clinical area is vital.* (Participant 42)<br><br>*I think promotion of your service, I think information sharing, that's what changes culture.* (Participant 41)	

	**Level of Implementation**			
	**High**		**Medium**	**Low**
	**Organisations**			
	**Organisation 4**	**Organisation 6**	**Organisation 17**	**Organisation 19**
Line management buy-in and support.	*The manager is very much around the whole survivorship programme. She really believes in it, and she believes in the benefit of it, but it does require you to have the time to send the staff off to be trained like we had to do.* (Participant 23)	*It comes from the CEO down and from our director of nursing down. I have gone to my director of nursing with things that are completely aside from survivorship and said to her 'I really want to do this, what do you think?' She'd be absolutely, tell me exactly what you need me to do, and I will 100% support you.* (Participant 04)	*It is such a pity, when I was looking, as part of the research I had to get the go-ahead from my director of nursing, she wouldn't even meet me about it, and she wouldn't sign the go-ahead for me.* (Participant 41)	*Suppose that's why you'll have your line manager and my line manager support. And that's as much as I need at the moment regarding it.* (Participant 05)

		I had built up a relationship with the Manager, but we're so under-resourced across the board, it was a big thing for her to say, 'Try it'. Here's some funding. I'll take you out of your clinical post.' So, someone had to fill my gap when I left, and I got this programme up and running. ... and her leadership and her support were key. (Participant 02)		

	Level of Implementation			
	**High**		**Medium**	**Low**
	**Organisations**			
**Codes**	**Organisation 7**	**Organisation 2**	**Organisation 17**	**Organisation 19**
Health provider and organisation priorities	*One of the biggest things that would have been said to me from very early on starting was that they want to develop something to help enhance cancer survivorship. So, it would have been from the get-go here. Overall, here, it would be positive and encouraging to create something to help with survivorship.*<br>(Participant 14)<br><br>*So, trying to have patients able to manage themselves at home, mainly for their quality of life...There's more of an emphasis*	*It's a natural extension of the work here.*<br>(Participant 18)	*In the acute setting we're putting out fires all the time, management aren't seeing the bigger picture, and the time and effort isn't going into that. If we keep these people well, know how to access things if they need them, the acute problems won't happen.*<br>(Participant 10)	*It's just the way we are set up. Survivorship is not number one, it's definitely down the list of priorities…higher management, their goals are more keeping clinic numbers down and keeping people out of A&E.*<br>(Participant 05)

	on admission avoidance first of all, well it is a hospital priority. (Participant 44)			

	**Level of Implementation**			
	**High**			**Medium**
**Organisations**				
**Codes**	**Organisation 4**	**Organisation 6**	**Organisation 2**	**Organisation 17**
SMS addressing organisation's goals	*How do we help people move on from the cancer centre, because it's not just about them coming into us, it's great to be able to support them. But we also have a responsibility to help them get on with their lives and move on from this and that's where the survivorship programme is very good.* (Participant 23)	*Because it decreases length of stay, there's more cost savings for the hospital, it was a neater business case, it was a nicer business case.* (Participant 02)	*It's a good way to help move patients on and out of the service. That's what you want. You want them to be able to self-manage.* (Participant 17)	*Management responds to data and numbers, that's how they work and function, so they want numbers in, numbers out, they don't want waiting lists. When you look for more resources, you have to come around to their language to explain to them and say, look this will ultimately reduce people coming into ED, that's where you have to sell it to them, but because you don't have strong data on immediate numbers they're not really interested or they don't understand it.*

				(Participant 41)
	**Level of Implementation**			
	**High**		**Medium**	**Low**
**Organisations**				
**Codes**	**Organisation 4**	**Organisation 7**	**Organisation 15**	**Organisation 19**
Incentives	*It's really important to acknowledge their contribution.* (Participant 36)	*I was part of the movement to make sure that there was an embedded payment process for leaders.* (Participant 20)	*She's highly acknowledged as well within our centre, and we'd always give her a donation, or a present, or a gift, a significant one to cover costs of coming every week, or a voucher, or something for herself.* (Participant 31)	*And there would be no extra, obviously no money for extra money for it (delivering programme) or anything like that. So that's definitely a barrier as well.* (Participant 05)

	**Level of Implementation**		
	**High**	**Medium**	
**Organisations**			
**Codes**	**Organisation 6**	**Organisation 10**	**Organisation 17**
Culture: Entrepreneurship	*There's a lot of very genuinely interested and motivated people who want (hospital) to be a centre of excellence and who are therefore very invested in providing and improving really good care. It feels like a very positive culture in that way. Often throughout the hospital, there'd be different innovation programs and things like that. So, I think the hospital culture is progressive, and it encourages development and innovation. There's an implied pressure to be doing more and to be delivering good care as possible. So it's a positive peer pressure element. And I think that's because the clinical governance, I think*	*The (hospital) is very supportive of things like this and kind of let you do what you want if it looks like a good idea. In other areas you might have to make a case for things a little bit more.* (Participant 38)	*We don't have performance meetings or anything like that, they don't want to know what we're doing as long as we're showing up for work, as long as we're practising safely, as long as there isn't a complaint about us...and it would be great if we had these performance meetings to say look, this is where I want to see, this is what I want to do. There's none of that, it's a pity that culture isn't there". (Participant 41)*

	*the governance is quite good. And for most people doing more, there's not too much, or at least I haven't experienced yet, too much bureaucratic pushback. If there is something that potentially could be developed, I think once people have a clear idea and want to do something, they're usually facilitated and supported in doing that. That's my experience. I think it is true that the culture promotes innovation. (*Participant 07*)*<br><br>*I do think it is a clinical environment that is motivated for change. (*Participant 04*)*		

	**Level of Implementation**			
	**High**	**Medium**	**Low**	
**Codes**	**Organisation 6**	**Organisation 10**	**Organisation 18**	**Organisation 20**
Hospital governance & ownership	*I've seen that throughout my training, I've worked in HSE direct funded hospitals, and in a couple of the voluntary hospitals, and I see a difference, to be honest. My impression, which is only an impression, I have absolutely no data on this, but my impression is that there's much more of a sense of ownership, and a sense of kind of responsibility and interest in making things as good as they can be, versus a sense of kind of executing*	*I've worked in HSE run hospitals which are quite different but the (hospital) has its own board of management and it tends to prioritise innovation over a lot of other things. So, we do have a certain amount of freedom in terms of how we design things, and the directorate is really, really supportive. Which really helps as well….I worked for four years in (hospital) which is the HSE directly run hospital and when I came to this (hospital) the difference is*	*Because most of them are voluntary, and they're not true HSE. So, you definitely can see the difference between voluntary hospitals and the HSE.* (Participant 06)	*I'm here a while now and things are just slower.* (Participant 40)

	what you were told to do, you know, kind of doing the requirements and having very little scope necessarily to push beyond that. Because I think in the HSE direct funded hospitals, you're working in a machine and you don't feel like you've got power, the capacities to even see that you could make change. Whereas when you're working in a hospital or a system that feels small and efficient, like that feels like you could try and make a change tomorrow and you'd be a step closer to it the day after you. You know, you might achieve it by the end of the year, that is much more motivating. So, I think there is a difference. (Participant 07)	*actually mind-blowing. We got a new nurse appointed and we had her in post within two weeks. In a HSE run hospital that would have taken two years. So, it just allows you to move that a little bit faster which is why we have our team up and running here.* (Participant 38)		
