# Supplementary material for: Contextual factors that influence adoption and sustainment of self-management support in cancer survivorship care: a practical application of theory with qualitative interviews
Source: BMJ Qual Saf. 2024 Nov 13;34(12):e017561. doi: 10.1136/bmjqs-2024-017561 (PMC12703244; doi:10.1136/bmjqs-2024-017561)
Supplement: online supplemental file 4 [file bmjqs-34-12-s004.pdf]

Supplementary file 4: Themes explaining the contextual factors influencing implementation with examples of supporting qualitative quotes.

**Policy is a driver of adoption, but infrastructure and resources in the inner setting are necessary for sustainment**		
**CFIR Construct**	**Codes**	**Sample quote(s)**
Policies & Laws	National policy	*I think the national strategy is definitely the main driver, that publishing of standards and requirements to roll out the survivorship care.* (Participant 01, Low Implementing Organisation).<br><br>*Broadly speaking if we look at policies and procedures and everything, there's been this paradigm shift that we're moving beyond this. It's not just about treating the cancer but now that we've got such an increase in survivorship, we're now starting to look at, OK, well what are the survivorship issues? (*Participant 07, High Implementing Organisation).
	National Programme	*And it's a national programme, so we wanted to be part of the national run of that programme as everybody else does in the country*. (Participant 09, High Implementing Organisation).
Financing	External financial support	*Initially through pilot funding and now the funding is secured. We applied for the feasibility funding, from the (Grant funder). That allowed us to test an intervention here.* (Participant 02, High Implementing Organisation).
Work infrastructure	Staffing arrangement and alignments with team responsibilities	*Now we have staffing confirmed and permanent moving forward. So that's been a really positive development.* (Participant 02, High Implementing Organisation)

	Competing Priorities	*I'm back late every time I do it, which doesn't work from a home point of view.* (Participant 24, Medium Implementing Organisation)
	Bureaucratic administrative tasks	*If there was more organisational support, certainly for me, if there was more organisational support from administration, I would probably be more inclined to do it.* (Participant 12, High Implementing Organisation)<br><br>*And they can help us with admin. So that works really well.* (Participant 20, High Implementing Organisation)
Available Resources	Funding at local level	*The money to resource is not there, that comes at a local level, and I think that's what would be needed to push this onto the next stage.* (Participant 01, Low Implementing Organisation).<br><br>*She had autonomy to be able to use her funding for something like this, which then obviously has led to the programme being developed further.* (Participant 02, High Implementing Organisation)<br><br>*There isn't equal access across the country because some places are very small, with very little funding behind them.* (Participant 23, High Implementing Organisation)
	Funding staff positions	*Usually, we have to have done some kind of pilot or shown the effectiveness of it but no matter how good that is, if we don't have a funding source, we can't get a post.* (*Participant 44*, High Implementing Organisation)<br><br>*So, we had that feasibility funding and then we had pilot funding from the hospital very short-term, and now there's been a post in cancer survivorship.* (Participant 02, High Implementing Organisation)
	Physical space	*We have the space, that's a big thing in the delivery of it, that's a huge thing.* (Participant 17, High Implementing Organisation)
	Time	*I have the time to do it*. (Participant 22, , High Implementing Organisation)<br><br>*It's so time consuming. There's a lot of work goes in behind organising it.* (Participant 17, High Implementing Organisation)

	Partnerships & connections	*We're able to do it with our local community and they can help us with admin. So that works really well.* (Participant 20, High Implementing Organisation)<br><br>*I'd be a big believer in connecting with other organisations within our community settings.* (Participant 36, High Implementing Organisation)<br><br>*There's no meeting space to deliver the group, so we have close links with (University), so the last time it was ran, we ran it in a room over in their building.* (Participant 03, High Implementing Organisation)
Mid-Level Leaders	Line management buy-in and support.	*The manager, she really believes in it. And she believes in the benefit of it, but it does require you to have the time to send the staff off to be trained like we had to do". (Participant 23, High Implementing Organisation)*<br><br>*I had built up a relationship with the Manager, but we're so under-resourced across the board, it was a big thing for her to say, 'Try it'. Here's some funding. I'll take you out of your clinical post.' So, someone had to fill my gap when I left, and I got this programme up and running. … and her leadership and her support were really key.* (Participant 02, High Implementing Organisation)<br><br>*It is such a pity, when I was looking, as part of the research I had to look for the go-ahead from my director of nursing, she wouldn't even meet me about it and she wouldn't sign the go-ahead for me, but I didn't let it stop me because there's a bigger picture to look at here.* (Participant 41, Medium Implementing Organisation)

**Accreditation, performance measurement and governance to enable adoption and continued engagement with implementation.**		
**CFIR Construct**	**Codes**	**Sample quote(s)**
Performance-Measurement Pressure	Accreditation	*We went for OECI accreditation and as part of that we had to formalise our structures and it's really come on from that then when we got the OECI accreditation. When we got that accreditation there was a Quality Improvement plan that went into place.* (Participant 44, High Implementing Organisation*)*

		The cancer services here have been realigned, so we got accreditation, to be a Cancer Centre. (Participant 11, High Implementing Organisation)
	Key performance indicators	*And whether that needs to be more prescriptive in our care, it needs to be measured by KPIs or through audit. I think something like that would motivate or enable better engagement. You need to see your reward for doing it.* (Participant 01, Low Implementing Organisation)<br><br>*So "KPI ticked", now we have this programme up and running and there is no way of feeding back into where it's going wrong.* (Participant 30, High Implementing Organisation)
Policies	Governance to guide monitoring and evaluation	*If you've got good quality management and good coordination and there's a feedback loop, that's very sustaining.* (Participant 20, High Implementing Organisation)<br><br>*I don't know where the governance lies of the course and who oversees the course evaluations, work improvement, quality management, all that stuff.* (Participant 30, High Implementing Organisation)

Providing evidence of SMS improving patient outcomes and addressing leadership priorities secures organisational buy-in			
**CFIR Construct**	**Codes**	**Sample quote(s)**	
Relative Priority	Health provider and organisation priorities	*It's just the way we are set up. Survivorship is not number one, it's definitely down the list of priorities…higher management, their goals are more keeping clinic numbers down and keeping people out of A&E.* (Participant 05, Low Implementing Organisation)<br><br>*It's fluffy and it seems like that it's a luxury to be able to deliver that as opposed to a necessity, it's an absolute necessity for patients.* (Participant 41, Medium Implementing Organisation)<br><br>*One of the biggest things that would have been said to me from very early on starting was that they want to develop something to help enhance cancer survivorship. So, it would have been from the get-go here. Overall, here, it would be quite positive to encouraging to create something to help with survivorship.*<br>(Participant 14, High Implementing Organisation)<br><br>*In the acute setting we're putting out fires all the time, management aren't seeing the bigger picture, and the time and effort isn't going into that. If we keep these people well, know how to access things if they need them, the acute problems won't happen.* (Participant 10, Medium Implementing Organisation)<br><br>*There's more of an emphasis on admission avoidance first of all, well it is a hospital priority.*<br>(Participant 44, High Implementing Organisation)	
Mission Alignment	SMS addressing organisation's goals	*Because it decreases length of stay, there's more cost savings for the hospital, it was almost a neater business case, it was a nicer business case.* (Participant 02, High Implementing Organisation)	

		How do we help people move on from the cancer centre, because it's not just about them coming into us, it's great to be able to support them. But we also have a responsibility to help them get on with their lives and move on from this and that's where the survivorship programme is very good. (Participant 23, High Implementing Organisation)<br><br>*The director definitely is a big fan. So, we see the benefit of it to patients and it's a good way to help move patients on and out of the service. That's what you want. You want them to be able to self-manage.* (Participant 13, Medium Implementing Organisation)<br><br>*Management responds to data and numbers, that's how they work and function, so they want numbers in, numbers out, they don't want waiting lists. When you look for more resources, you have to come around to their language to explain to them and say, look this will ultimately reduce people coming into ED, that's where you have to sell it to them, but because you don't have strong data on immediate numbers they're not really interested, or they don't really understand it.* (Participant 41, Medium Implementing Organisation)	
	Individual healthcare provider goals and priorities	*If you're surgical you're going to look at your post op complications and your length of stay, if you are an oncologist, you're going to look at the survival rates and the quality of life. Whereas if you're like me or social workers you're looking at quality of life and function.* (Participant 06, Low Implementing Organisation)	
Innovation Source	Health professional led	*You won't get medical buy in unless there are medical people doing it*. (Participant 16, High Implementing Organisation)	
	Consultant endorsed	*You really don't know where to go with hospital management. We have yet without the support of the consultants, and really the badgering from the consultants that they really need this to make their service work. Unless the consultant is leading it out for a patient and really recommending and supporting it, it becomes a challenge, then you're on an uphill battle to try and engage people.*<br>(Participant 01, Low Implementing Organisation).	
Innovation Evidence-Base	Evidence of effectiveness	*From all the research that's been done behind the programme that has been proven to work in that way.* (Participant 29, High Implementing Organisation)<br><br>*It was very much about allowing the organisation to provide programmes that have research done into them, that have a proven track record. I felt for an organisation that was so small, I was really conscious of ensuring*	

		I pointed the organisation towards what I felt were sustainable deliverable types of programmes that had a proven benefit to patients. (Participant 36, High Implementing Organisation)	
	Testimonials	*But once they had a couple of patients go through it, and they got feedback from their patients, they're very happy to refer. Again, because it's safe, because it's evidence based.* (Participant 20, High Implementing Organisation)<br><br>*The feedback from clients There was very little, very little negative, there was nothing really negative about the contents of the programme.* (Participant 19, High Implementing organisation)<br><br>*I wasn't sure about the programme, but after delivering it's powerful…I took a look at the summaries of evaluations and it's so consistent.* (Participant 13, High Implementing organisation)	
	Local data	*Data is everything – if you want something to progress, you need the numbers, because it decreases length of stay, there's more cost savings for the hospital.* (Participant 42, Medium Implementing Organisation)<br><br>*It's the local data that motivates higher management to do it. Having local data, that has an impact where you can show what is happening on the ground. That is worthwhile and hard to argue with.* (Participant 47, High Implementing Organisation)	
Reflecting & evaluating: innovation	Information about the success of the program.	*I'd love to see a national review of what we've done so far. And how that has impacted people because we don't really know, we do our own review, you know, and they're always really well evaluated I think. We really need to know, nationally, how are we doing here? How well we've done or what impact these programs have.* (Participant 09, High Implementing Organisation).<br><br>*To see the validity in the program, they have to see it running within the community and get feedback.* (Participant 36, High Implementing Organisations)<br><br>*To keep it going they need to see the statistics that people are taking part.* (Participant 24, Medium Implementing Organisations)	
Relative advantage	Overshadowed by new priorities	*The (SMS programme). There's still a lot of work to be done to promote it. You know, I follow all these groups on Twitter and it's as if then they kind of pick a thing that they're going to promote. At the minute, it's all the children and young adults coming through on my timeline, absolutely, that has to be promoted. But I haven't seen much of the (SMS programme) coming through. I think there's a lot of work to be done there to promote it.* (Participant 21, High Implementing Organisation)	

	Advertising of the programme	*When it comes to sustainment, you need to continue marketing it and you need to continue advertising it. I think if you want a program like this to continue, you need to continue advertising it to people. You need to bring in people that have done it and for them to say it 'I got this benefit out of it'.* (Participant 26, Medium Implementing Organisation)	

**Champions with proactive leadership and entrepreneurial skills enable adoption and sustainment**		
**CFIR Construct**	**Codes**	**Sample quote(s)**
Implementation leads	Bottom-up drive and commitment	*Now we have staffing confirmed and permanent moving forward. So that's been a really positive development, but that came from ourselves in the social work department, as opposed from a higher management point of view who pushed it.* (Participant 15, Medium Implementing Organisation)<br><br>*I'm the driver in the service, I'm the one who keeps pushing, pushing, pushing, pushing, because I've been in the service so long, I understand the way it works, but I have a vision, I might not get it done today or tomorrow and I might be knocked down today and tomorrow, but I'll keep going, there's always solutions. So, you're forced to work within the structures that you have but I'll keep asking so it's not going to stop me that I'm getting 'no'. I'll keep asking and I'll keep putting forward my business case.* (Participant 41, Medium Implementing Organisation)
	Respected	*Because (champion in another high implementing organisation) was there from the very start pushing. And she's so good at what she does, she was so highly respected, that she could literally walk into a meeting with anyone and sell them ice. Just brilliant, which is the way we need to be. But it's a shame that we need to be like that to get….and shaping the way things are.* (Participant 06, Low Implementing Organisation)<br><br>*I really think she's a doer, and she likes to do things well, and she likes to think properly. And because she has such a wealth of experience, people come to her, she's a known figure. She is keen to continue to improve and develop things.* (Participant 07, High Implementing Organisation).

	Entrepreneurialism	*She has been great for grants and funding. So, without that, without us getting that grant there still would be no survivorship.* (Participant 10, Medium Implementing Organisation)
	Personal interest	*From a personal level is that I had a personal interest in oncology, and I had previously completed self-management research. It came nationally, internationally, and then personally with my own drive to develop the intervention.* (Participant 03, High Implementing Organisation)<br><br>*Nobody has come to me and said you need to do this; this is what we have chosen, and this needs to be part what you're delivering if you're delivering a survivorship service.* (Participant 21, High Implementing Organisation)
	Communication to develop connections, leverage networks and secure buy-in	*I think part of the reason is because of how I did it. I'm one of those people who you can tell talks a lot. So, I brought everybody on the journey with me. The admin people knew all about this before they ever knew what their role was going to be. My colleagues, my nursing colleagues knew all about it before they even knew where they were going to fit into this.* (Participant 04, High Implementing Organisation)<br><br>*My strategy really was to keep talking about it to every single person.* (Participant 20, High Implementing Organisation)
	Championing qualities	*Health professionals who are already overstretched try to do this as well. And I think that's when it's dropped. It's only those of us that really stick it out have continued with it.* (Participant 12, High Implementing Organisation)<br><br>*I choose to do it outside of my working day. So that's a personal choice for me, one really good thing about the programme from my perspective, is that it's a really good adjunct to the work that I do.* (Participant 20, High Implementing Organisation)<br><br>*I think individuals can do things if they're motivated enough and can manage their time…but that's then at a cost to other members of the team picking up some of your work.* (Participant 42, Medium Implementing Organisation)

**Organisational culture of entrepreneurship and addressing employee well-being affects the capacity of champions and staff to adopt and sustain programmes**		
**CFIR Construct**	**Codes**	**Sample quote(s)**

Culture: Learning-Centeredness	Culture of innovation & entrepreneurship	*There's a lot of very interested and motivated people who want (hospital) to be a centre of excellence and providing and improving really good care. It feels like a very positive culture in that way. So, I think the hospital culture is progressive and it encourages development and innovation. There's an implied pressure to be doing more and to be delivering good care as possible. And I think that's because the clinical governance is quite good. And for most people doing more, there's not too much, or at least I haven't experienced yet, too much bureaucratic pushback if there is something that potentially could be developed. I think once people have a clear idea and want to do something, they're usually facilitated and supported in doing that. I think it is true that the culture promotes innovation.* (Participant 07, High Implementing Organisation)<br><br>*We don't have performance meetings or anything like that, they don't want to know what we're doing as long as we're showing up for work, as long as we're practising safely, as long as there isn't a complaint about us...and it would be great if we had these performance meetings to say look, this is where I want to see, this is what I want to do. There's none of that, it's a pity that culture isn't there.* (Participant 41, Medium Implementing Organisation)
	Administrative organisational structures	*The dynamic, so it's very hard, there's layers of management, so it's very hard to get an actual appointment with our director of nursing, it's very hard –we're supposed to go through the proper channels to try and agree anything, so if I am to ask my director of nursing to request on my behalf that I get clerical support, well she's going to business managers, but any motion you put through to speak directly to the director of nursing about it, it could take a year for anything to happen, it is so arduous, all these executive council meetings, they bring up different topics and nothing, nothing is done .. you can only have these meetings every three months, came back again, right and then nothing.* (Participant 41, Medium Implementing Organisation)<br><br>*That's where you lose the motivation to push things forward, because it takes so long, you're motivated but that motivation starts to wear off, as time passes and there's no progress. You could be chasing emails, like what are we doing next, are we meeting again....you're putting energy onto something that's not progressing". (Participant 42, Medium Implementing Organisation).*

	Hospital governance and ownership	*They're supportive of things. If it looks like a good idea, in other areas you might have to make a case for things a little bit more and I've worked in HSE run hospitals which are quite different but the (hospital) has its own board of management and it tends to prioritise innovation over a lot of other things.* (Participant 38, Medium Implementing Organisation)<br><br>*I've worked in HSE direct funded hospitals, and in a couple of the voluntary hospitals, and I see a difference, it's my impression, which is only an impression, I have absolutely no data on this, but my impression is that there's much more of a sense of ownership, and a sense of responsibility and interest in making things as good as they can be, versus a sense of executing what you were told to do, doing the requirements and having very little scope necessarily to push beyond that. .* (Participant 07, High Implementing Organisation)
Culture: Deliverer-Centeredness	Non-monetary incentives	*It's really important to acknowledge their contribution. So, we nominated our peer leader here for an award last year. One of the things was her work on the SMS programme. So, it's important to acknowledge it.* (Participant 36, High Implementing Organisation)<br><br>*It doesn't have to be monetary, but some appreciation of the time and all that.* (Participant 22, High Implementing Organisation)
	Supervision	*We started supervision. So that was very important, and our director of services recognised that and how much you could be holding. So, for managing our boundaries that helped greatly. That we could go somewhere with that after the programme, because it brings up a lot of emotional issues for clients. That was a very good strategy on the on our director of services part.* (Participant 19, High Implementing Organisation)<br><br>*Supervision has always been part of our practice, but I don't know if it is for a lot of the other professions in this area….there's a lot of an emotional load that comes with working in oncology, and I think if people are to be sustained in the work, and if people are also to keep their warmth and care towards patients, they have to be supported themselves as well.* (Participant 15, Medium Implementing Organisation)
	Debriefing	*I realised it was good just after the session that you can debrief and talk directly about the session.* (Participant 32, High Implementing Organisation)<br><br>*I think the debriefing is really important, because if you're not a trained psychologist, I'm really lucky in that sense I'm trained, and I know how to manage it. And I think it's a lot to hear everybody's story, the sadness around that is really hard to hold if you're not trained in that way.* (Participant 33, High Implementing Organisation).

	Continuous professional development certifications	*In terms of the professionals could this stand to their professional development, continuing development credits.* (Participant 28, High Implementing Organisation).
Teaming	Bringing team members together	*Really for the centre to take it on and to do it effectively, this requires a lot of training around each session. So, for example, before I do each session, I would meet with my volunteer the day before, or a number of days before, and we go through each session, we practice that. You have to make sure the volunteers are comfortable of course. I have a new volunteer just trained this year so that can take up a lot of time that maybe isn't captured.* (Participant 19, High Implementing Organisation)
Engaging Deliverers	Engaging	*I'm a volunteer with them, but I'm very much part of the team there, and I feel connected and that's to do with the management of the centre and the way they hold their volunteers. So, it's just the way the centre holds us and keeps engaging with us and checking in with us and so forth. So, I feel very much part of the team there.* (Participant 29, High Implementing Organisation)
